# Carry out case-based scenario teaching in medical students to improve skills of occupational protection against infectious diseases

**DOI:** 10.1017/ash.2025.128

**Published:** 2025-09-03

**Authors:** Zhao Xia, Zhao Huijie, Yang Yang, Zhao Zhongjing, Ma Wenhui, Hanxu, Wang Yuncong, Wang Wei

**Affiliations:** 1 Xuanwu HospitalCapital Medical University, Beijing, 100053

**Keywords:** Occupational Protection, infectious Diseases, Case-based Scenario Teaching, Medical Students

## Abstract

**Objective:** Optimized teaching methods in medical students to improve skills of occupational protection against infectious diseases and reduce the risk of developing infectious occupational exposure in clinical practice. **Methods:** Establish a database of infectious occupational exposure cases in clinical practice based on monitoring data. Teaching guided by cases and videos-based scenario was carried out in the experimental group and traditional theoretical teaching was carried out in the control group in medical students. And then conducted a questionnaire survey on knowledge and skills of occupational protection against infectious diseases and observed the frequency and the prescriptive disposal measures of infectious occupational exposure in clinical practice in two groups. **Results:** The infectious occupational exposure database included a total of 95 typical cases in 6 categories, including various sharp weapon injuries and mucosal exposure. There were 116 medical students involved in the study across the course of 12 months. The incidence of infectious occupational exposure in medical students during clinical practice internships was 18.9%. Compared with the control group, the awareness rate of knowledge and skills of occupational protection against infectious diseases significantly increased (91.8% vs 87.0%, P<0.05), the incidence of infectious occupational exposure during clinical internships has decreased (15.6% vs 23.1%, P<0.05), and the implementation rate of prescriptive disposal measures after exposure has increased (91.7% vs 83.3%, P<0.05) in the experimental group in medical students. **Conclusion:** The case-based scenario teaching in medical students improved skills of occupational protection against infectious diseases and decreased the incidence of infectious occupational exposure during clinical internships. The effect of the optimized teaching methods was significant which is recommended to carry out widely.

